# Molecular Impact of the Tumor Microenvironment on Multiple Myeloma Dissemination and Extramedullary Disease

**DOI:** 10.3389/fonc.2022.941437

**Published:** 2022-07-01

**Authors:** Stefan Forster, Ramin Radpour

**Affiliations:** ^1^ Tumor Immunology, Department for BioMedical Research (DBMR), University of Bern, Bern, Switzerland; ^2^ Department of Medical Oncology, Inselspital, Bern University Hospital, University of Bern, Bern, Switzerland

**Keywords:** multiple myeloma, tumor microenvironment, TME, targeted therapy, malignant plasma cells

## Abstract

Multiple myeloma (MM) is the most common malignant monoclonal disease of plasma cells. Aside from classical chemotherapy and glucocorticoids, proteasome inhibitors, immunomodulatory agents and monoclonal antibodies are used in the current treatment scheme of MM. The tumor microenvironment (TME) plays a fundamental role in the development and progression of numerous solid and non-solid cancer entities. In MM, the survival and expansion of malignant plasma cell clones heavily depends on various direct and indirect signaling pathways provided by the surrounding bone marrow (BM) niche. In a number of MM patients, single plasma cell clones lose their BM dependency and are capable to engraft at distant body sites or organs. The resulting condition is defined as an extramedullary myeloma (EMM). EMMs are highly aggressive disease stages linked to a dismal prognosis. Emerging literature demonstrates that the dynamic interactions between the TME and malignant plasma cells affect myeloma dissemination. In this review, we aim to summarize how the cellular and non-cellular BM compartments can promote plasma cells to exit their BM niche and metastasize to distant intra-or extramedullary locations. In addition, we list selected therapy concepts that directly target the TME with the potential to prevent myeloma spread.

## Introduction

Multiple Myeloma (MM) is a hematologic malignancy defined by the expansion of a clonal plasma cell population within the bone marrow (BM) that results in the manifestation of clinical symptoms such as bone pain, pathologic fractures and anemia (1). In almost all MM patients, disease development is characterized by a distinct sequence of primary molecular alterations leading to the early transformation of a plasma cell into its malignant counterpart. The acquisition of additional secondary mutations results in further disease progression. In addition, the tumor microenvironment (TME) and treatment-related selection mechanisms promote the expansion of distinct plasma cell clones with the most aggressive phenotype (2, 3). According to the international myeloma working group, the diagnosis of MM requires the presence of ≥ 10% clonal BM plasma cells or a biopsy-proven extramedullary plasmacytoma combined with at least one myeloma defining event (e.g., evidence of end-organ damage) and positive detection of defined biomarkers (4).

In the last decade, new MM therapy options have substantially progressed. Combination of standard chemotherapy and glucocorticoids with proteasome inhibitors (PI), immunomodulatory drugs and monoclonal antibodies (mAb) as well as other substances with innovative mechanisms of action have evidently improved the outcome of many MM patients (5). However, despite the fundamental achievements in the understanding of MM pathogenesis and the continuous supply of novel therapies, almost all MM patients eventually suffer from disease relapse (6, 7).

**Figure 1:**
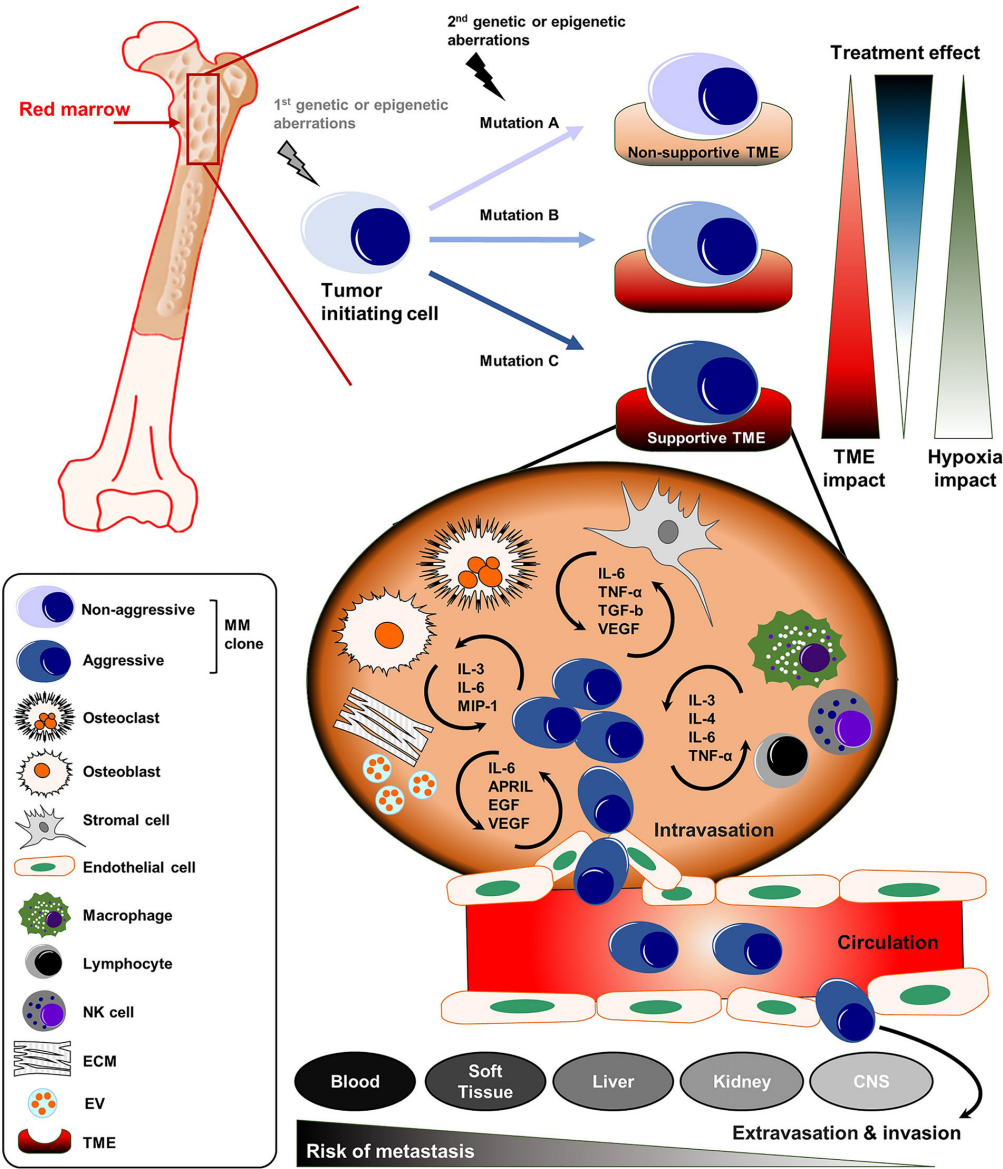
The complex organization of cancer initiation, progress, and distant metastasis of MM. MM is defined by the clonal expansion of a malignant plasma cell population upon genetic/epigenetic aberrations within the bone marrow (BM). The acquisition of secondary mutations in MM leads to further generation of sub-clones with a more aggressive phenotype. The dynamic influence of the TME and treatment-related mechanisms drive the progression of MM into even more aggressive disease stages. The malignant plasma cell clones can disseminate into the blood or to distant body sites or organs including soft tissue, liver, kidney and CNS. APRIL, a proliferation-inducing ligand; CNS, central nervous system; ECM, extracellular matrix; EGF, epidermal growth factor; EV, extracellular vesicle; IL, Interleukin; MIP-1, macrophage inflammatory protein 1; MM, multiple myeloma; NK, natural killer; TGF-b, transforming growth factor beta; TME, tumor microenvironment; TNF-α, Tumor necrosis factor alpha.

Refractory or relapsed disease stages bear a fundamental risk for further disease progression into the highly aggressive MM end-stages extramedullary myeloma (EMM) and plasma cell leukemia (PCL) (8–11). Here, the malignant plasma cell clones are no longer dependent on the BM microenvironment (as their extrinsic support). They are able to survive and engraft at distant body sites or organs including soft tissue, skin, liver, kidney and the central nervous system (CNS) (**Figure 1**) (8, 12, 13). In around two-thirds of patients suffering from EMM, clonal plasma cells break through the cortical bone and infiltrate the adjacent soft tissue. In the remaining one-third, hematogeneous dissemination to distant sites or organs can be observed (14). Patients with EMM and PCL have a significantly shorter overall survival and sufficient therapy options are often limited (8, 14, 15).

The tumor microenvironment (TME) is composed of different cellular compartments (e.g., endothelial cells, stromal cells, osteoclasts, osteoblasts and immune cells) and non-cellular fractions including the liquid milieu (e.g., growth factors, chemokines and cytokines) or the extracellular matrix (16). Emerging literature shows that the TME plays a key role in promoting myeloma survival, drug resistance and disease dissemination (**Figure 1**) (17). Preventing the infiltration and spread of myeloma cells to sites where they are capable to turn into quiescent states or colonize niches that are less accessible for standard therapies might play a significant role in overcoming EMM. In addition, therapies that can target or modulate the dynamic interactions of tumor cells with the TME, using different approaches such as immunotherapy or specific tyrosine kinase inhibitors, could overcome resistance and prevent the spread/metastasis of different solid and non-solid cancer entities including MM (**Table 1**) (18–20). In this review, we address the role of the TME on myeloma progression and dissemination and discuss potential therapies that could specifically target the TME to prevent manifestation of extramedullary disease (EMD).

**Table 1:** Overview of therapy approaches targeting TME compartments in MM and related clinical trials.

Compartments	Drugs	Mechanism of action	Clinical phase	Number of participants	Disease	Status	NCT Number
Osteoblasts/Osteoclasts	OTX015/MK-8628/Birabresib	BET-Inhibitor	Phase I	141		Completed	NCT01713582
	Denosumab	Anti-RANKL	Clinical approval	–	–	Clinical approval	–
	BHQ880	Anti-DKK1	Phase I	28	RRM	Completed	NCT00741377
	Zoledronic acid	Bisphosphonates	Clinical approval	–	–	Clinical approval	–
Bone marrow stromal cells	BMS-936564/MDX1338/Ulocuplumab	Anti-CXCR4	Phase I	46	RRM	Completed	NCT01359657
	AMD3100/Plerixafor	CXCR4 antagonist	Phase I/II	58	RRM	Completed	NCT00903968
	Masitinib (AB1010)	Multi-TKI (FGFR inhibitor)	Phase II	24	RRM with t(4;14)	Completed	NCT00866138
	CNTO 328/Siltuximab	Anti-IL6	Phase II	307	RRM	Completed	NCT00401843
	AVE1642	Anti-IGF1R	Phase I	26	Advanced MM	Completed	NCT01233895
Endothelial Cells	Bevacizumab	Anti-VEGF mAb	Phase II	102	RRM	Completed	NCT00473590
	BI-505	Anti-ICAM-1	Phase I	35	RRM	Completed	NCT01025206
	BG00002/Natalizumab	Anti-α4β1/Anti-VLA4	Phase I/II	6	RRM	Terminated	NCT00675428
Tumor-associated macrophages	BI 765063	Anti-SIRPα	Phase I	116	Advanced solid tumors	Recruiting	NCT03990233
	CNTO 888/Carlumab	Anti-CCL2	Phase I	44	Solid tumors	Completed	NCT00537368
	Zoledronic acid	Bisphosphonates	Clinical approval	–	–	Clinical approval	–
Hypoxia	Evofosfamide (Evo or TH302)	Hypoxia-activated prodrug	Phase I/II	98	RRM	Unknown	NCT01522872

BET, Bromodomain and Extra-Terminal motif; CCL2, C-C Motif Chemokine Ligand 2; CXCR4, C-X-C Motif Chemokine Receptor 4, DKK1, Dickkopf-1; FGFR, fibroblast growth factor receptor; ICAM-1, intercellular adhesion molecule-1; IGF1R, insulin-like growth factor 1 receptor; IL6, Interleukin 6; MM, multiple myeloma; RANKL, Receptor activator of nuclear factor kappa-B ligand; RRM, relapsed/refractory myeloma; SIRPα, Signal regulatory protein α; TKI, tyrosine kinase inhibitors; VEGF, vascular endothelial growth factor; VLA4, Very Late Antigen-4.

## Cellular Compartments of the TME

### Bone Marrow Stromal and Vascular Endothelial Cells

The C-X-C motif chemokine ligand 12-C-X-C motif chemokine receptor 4 (CXCL12-CXCR4) interaction is one of the earliest steps regulating plasma cell homing to the BM. Bone marrow stromal cells (BMSCs) and vascular endothelial cells produce the chemoattractant stromal cell derived factor 1 alpha (SDF-1α/CXCL12) that binds to CXCR4 resulting in homing and/or retention of hematopoietic stem and progenitor cells to the BM (21, 22). The CXCL12/CXCR4 axis has been indicated to regulate cancer cell invasion and metastasis in different solid cancer entities such as breast cancer (23, 24). In MM, high CXCR4 expression levels are associated with a worse prognosis and enhanced risks for EMD development (25). To unravel the effect of CXCR4 on myeloma cell dissemination, Roccaro et al. intravenously injected the human myeloma cell line MM.1S into immunodeficient NOD/SCID gamma (NSG) mice to generate clones that primarily engrafted in the BM (BM-prone clones) and clones that survived outside the BM (EMD-prone clones). Comparative analysis between both populations showed higher CXCR4 expression levels, enhanced epithelial-to mesenchymal transition and higher properties for extramedullary dissemination in EMD-prone clones (26). Treatment with the anti-CXCR4 monoclonal antibody ulocuplumab (MDX1338) resulted in reduced homing and engraftment of injected MM.1S cells (22, 26). In contrast, treatment with the PI bortezomib and the immunomodulatory agent thalidomide have been reported to induce the downregulation of both molecules, CXCR4 and CXCL12, which could provoke the dissemination of plasma cell clones (27–29).

Additionally, other surface molecules are associated with myeloma dissemination. Vascular cell adhesion molecule 1 (VCAM-1) is expressed on both BMSCs and endothelial cells. The VCAM-1 ligand, very-late antigen 4 (VLA-4), is frequently overexpressed on malignant plasma cells in the BM and plays an important role in plasma cell homing and cell adhesion (30, 31). In this regard, Hathi et al. showed that CRISPR/Cas9 induced depletion of VLA-4 in murine 5TGM1 myeloma cells resulted in increased extramedullary spread to soft tissue sites, liver and spleen and to reduced intramedullary tumor burden compared to mice injected with 5TGM1-wildtype cells where no manifestation of EMD occurred (32). In line with these findings, heparanase-induced shedding of CD138 (Syndecan-1), expressed on normal and malignant plasma cells, promotes myeloma cell migration and invasion by coupling VLA-4 to the vascular endothelial cell growth factor receptor-2 (VEGFR2) (33). Loss of CD56 or downregulation of CD44, two molecules involved in the adhesion of myeloma cells to BMSCs and endothelial cells, are frequently observed in advanced and disseminated disease states (34–38). Platelets, produced by megakaryocytes in the BM, have been found to protect disseminated cancer cells from immune attack and shear stress in the blood stream (39, 40). Platelet-cancer cell interactions further facilitate the extravasation and engraftment of disseminated cancer cells at distant body sites (39, 41, 42). Co-incubation of the myeloma cell line OPM-2 with platelets promoted proliferation and resulted in increased rates of BM engraftment compared to untreated controls (42). Roundabout Guidance Receptor 1 (ROBO1) is another molecule involved in the adhesion of myeloma cells to BMSCs. ROBO1 knock-out in myeloma cell lines resulted in impaired engraftment and extramedullary dissemination in immunodeficient mice (43).

Besides direct cell-cell interactions, BMSCs and endothelial cells produce and secrete a variety of soluble factors such as interleukin 6 (IL-6), insulin-like growth factor-1 (IGF-1), vascular endothelial growth factor (VEGF) and fibroblast growth factor (FGF) that are all reportedly linked to myeloma progression and cancer cell dissemination. Those soluble factors are promising targets for antibody-based therapy approaches tested in phase I and II clinical trials (**Table 1**) (44, 45). Therapy-and/or cytokine mediated disruption of ligand-receptor interactions that are involved in plasma cell homing or adhesion might increase the number of cancer cells invading the blood circulation (intravasation). Especially at late disease stages, where single cell clones might harbor the capacity to survive outside the BM microenvironment, the retention of cancer cells in the blood circulation might result in a leukemic spread or enable cells to engraft at distant body sites to form extramedullary tumor foci.

### Tumor Infiltrating Lymphocytes

Recognition and elimination of dysfunctional cells is a pivotal task of the adaptive immune system, especially of CD8^+^ cytotoxic T lymphocytes (CTLs) (46). Upregulation of programmed cell death-ligand 1 (PD-L1) on cancer cells inhibits immune attack *via* binding to its receptor, programmed cell death protein 1 (PD1), expressed on activated T cells (47). High PD-L1 expression levels have been observed on malignant plasma cells making it a promising target for immune checkpoint inhibitors (ICIs) (18, 48, 49). In addition, enhanced numbers of PD1 positive and T cell immunoglobulin and mucin domain-containing protein 3 (TIM3) positive T cells were detected in the BM of MM patients suggesting reduced T cell activation (T cell exhaustion) and an immunosuppressive environment (50). However, up to now, ICI monotherapies have failed to show clinical benefits in myeloma patients (51). The combination of ICIs with the immunomodulatory agent pomalidomide even resulted in a higher risk for adverse or toxic side effects in phase III clinical trials (52).

Dendritic cells (DCs) in the TME might additionally protect myeloma cells from CTL mediated cell killing. Studies by Leone et al. demonstrated that myeloid DCs can downregulate the expression levels of proteasome subunits in myeloma cells thus preventing CTL induced elimination (53). Recent studies have shown promising concepts overcoming the limitations of ICI based therapies in MM. Exemplary, the combination of ICIs with anti-CD38 monoclonal antibodies has been tested in pre-clinical studies showing encouraging results (54). Though, further research is needed to elucidate the mechanisms of ICI failure in MM and to provide novel therapies that increase the efficacy of immune checkpoint blockade in myeloma patients.

### Tumor-Associated Macrophages

Tumor-associated macrophages (TAMs) are known to promote disease progression in numerous solid malignancies including glioblastoma, melanoma, colorectal cancer, lung cancer or ovarian cancer (55–59). Emerging evidence highlights their contribution in driving dissemination of myeloma cells within the skeletal system and to extra-medullary sites. A variety of pre-clinical models has been used to investigate how TAMs affect myeloma cell survival and dissemination. Diphtheria-toxin mediated abrogation of tissue-resident CD169^+^ macrophages in CD169-DTR mice, intratibially injected with myeloma cells from a syngeneic Vk*MYC mouse model, resulted in reduced dissemination of myeloma cells into the blood circulation and to the contralateral tibial bone. Mechanistically, the pro-inflammatory cytokines IL-6 and tumor necrosis factor alpha (TNFα) secreted by CD169^+^TAMs, increase vascular leakage and downregulate CD138-mediated cell adhesion; thus, driving myeloma intravasation (60). Concomitantly, M2 polarized macrophages are known to promote angiogenesis (61). Reduced blood vessel density and VEGF expression levels were observed in myeloma xenografts injected with M2-polarized macrophages and treated with the macrophage-depleting agent clodronate. In contrast, xenografts in untreated mice showed enhanced tumor growth and increased VEGF blood concentrations (62). In line with these findings, transcriptomic profiling of the immune cell compartment in patients suffering from refractory or relapsed myeloma, revealed enrichment of a distinct subset of macrophages expressing genes linked to angiogenesis such as VEGF-A or diphtheria toxin receptor (Heparin-Binding EGF-Like Growth Factor) (63). BMI1, a member of a polycomb group multiprotein complex, is overexpressed in macrophages of 5T-myeloma mice compared to macrophages derived from healthy mice. Macrophages, isolated from myeloma bearing 5T BMI1-knock out mice showed reduced production of the pro-angiogenic factors VEGF and nitric oxide (NO). Besides their impact on angiogenesis, TAMs exert direct and indirect effects on myeloma cells preventing detection and elimination by the immune system or enhancing myeloma cell survival and drug resistance.

*In vitro* co-culture experiments using myeloma cells and macrophages revealed that the presence of M2 macrophages can prevent myeloma cells from undergoing apoptosis induced by bortezomib or melphalan treatment; hence, increasing resistance and promoting progression (64, 65). In addition, Wang et al. reported that high numbers of CD163^+^ TAMs within the tumor infiltrating immune cell population of myeloma foci are associated with a dismal outcome in myeloma patients receiving bortezomib-based treatment regimens (66). Reduced CXCR4, as a homing receptor for normal and malignant plasma cells in the BM, was detected in myeloma patients undergoing bortezomib-treatment. CXCR4 downregulation is reportedly linked to impaired adhesion and high levels of the macrophage migration inhibitory factor (MIF) (27, 67). Upregulation of CD47 expression can be observed in around 70% of MM patients. Binding of CD47 to signal regulatory protein α (SIRPα) on macrophages acts as a “don’t eat me signal” and protects myeloma cells from phagocytosis and apoptosis (68). Antibodies targeting CD47 or SIRPα are currently tested in clinical trials (**Table 1**).

In summary, TAMs might drive the extra-medullary expansion of myeloma cells from the BM into the blood circulation *via* stimulation of blood vessel formation and a cytokine-mediated increase of the vessel-wall permeability. TAMs additionally exert direct and indirect effects on neighboring myeloma cells driving the downregulation of important adhesion molecules such as CD138 and CXCR4. Pro-inflammatory cytokines such as IL-6 and TNFα that are released by TAMs, finally enhance cell migration and tissue invasion favoring cancer cell dissemination (60). Lastly, drug-treatment based selection mechanisms might generate myeloma cell clones that can survive without the essential factors provided by the BM microenvironment and gain the ability to engraft at different body sites and organs.

### Osteoblasts and Osteoclasts

In contrast to TAMs or BMSCs, the impacts of osteoblasts and osteoclasts on myeloma cell dissemination are less understood. In MM patients, over-activation of osteoclasts and inhibition of osteoblastic differentiation often result in a dis-equilibrium that promotes resorption of healthy bone material and impairs osteoblastogenesis (69–71). As a consequence, myeloma patients frequently experience bone pain and spontaneous fractures that significantly affect morbidity and reduce quality of life (72, 73). Osteoclasts have been found to influence myeloma progression *via* direct crosstalk or release of distinct cytokines including IL-6, IL-3, macrophage inflammatory protein 1 alpha (MIP1α) or expression of receptor activator of NF-κB ligand (RANKL) (74). Expression of RANKL and secretion of IL-6, MIP1α or IL-3 by myeloma cells activates osteoclasts and thereby increases resorption of bone matrix (74–78). Enhanced resorption of bone material results in the release of different soluble factors such as IL-6, BAFF or APRIL that stimulate myeloma growth in a feed-forward mechanism also known as the “vicious cycle” (78, 79). IL-6 promotes myeloma cell dissemination *via* downregulation of CD138 expression and increases the vessel wall permeability; therefore, facilitating intravasation of cancer cells (60). In addition, osteoclasts can activate dormant myeloma cells in the BM, thereby, initiating micrometastases and provoking disease relapse (80). While osteoclasts have a pro-tumorigenic role, osteoblasts counteract osteoclast activity by keeping myeloma cells in quiescent cell states and *via* induction of apoptosis (80). For this reason, myeloma cells inhibit osteoblasts through the release of distinct secretory factors such as dickkopf-related protein 1 (DKK1), secreted frizzled-related protein 2 (sFRP2) and transforming growth factor beta (TGFβ) to overcome the tumor-suppressive effects of osteoblasts (76, 81, 82). In Phase I clinical trials, anti-DKK1 antibodies (BHQ880) were tested for their potential to overcome the myeloma-mediated inhibition of osteoblasts (83). In addition, preclinical *in vivo* studies using genetically modified C57BL6/KaLwRij mice with RUNX family transcription factor 2 (RUNX2) deficient osteoblasts indicate that RUNX2 deficiency in MM osteoblasts, triggers myeloma cells and enhances myeloma dissemination and growth (84).

## Non-Cellular Compartments of the TME

Direct cell-cell interactions and soluble factors that are released by the TME are important stimuli that influence engraftment and dissemination of myeloma cells. Aside, additional TME related mediators have been found that can promote myeloma dissemination. Lack of nutrients with changes in the cell metabolism and/or increased hypoxic tension, influence the surface expression of distinct cell surface molecules on plasma cells (PCs) thereby favoring a pro-migratory phenotype with enhanced dissemination properties. In this regard, CD138 negative malignant PCs that had been isolated from tumor bearing Vk*MYC mice showed higher rates of migration, cancer cell dissemination and intravasation when injected into syngeneic recipients. In contrast, a high CD138 expression was associated with enhanced rates of tumor growth. Starvation experiments showed that CD138 is dynamically controlled by the availability of serum-components provided by the BM environment and that loss of CD138 upon starvation could be partially restored after addition of serum (85). In this regard, a high tumor burden with low nutrient levels might induce CD138 downregulation thus promoting single cell intravasation. In the blood stream with enhanced nutrient availability, PC might regain CD138 expression and exit the circulation to start another round of engraftment and growth. Besides, CD138 and CXCR4 expression levels are also dependent on the oxygen levels present in the BM. Especially at advanced and high-risk MM stages high rates of cancer cell proliferation might increase the consumption of nutrients and oxygen within the BM. In this regard, hypoxia has been shown to reduce CD138 and CXCR4 expression driving myeloma cells into an immature/stem-cell like state with enhanced properties for dissemination (86, 87).

Extracellular vesicles (EVs) are cellular particles released by all different types of cells and have been proposed to play an important role in the pathogenesis of EMD (88, 89). Different mechanisms have been described of how EVs can promote MM progression and myeloma cell dissemination. EVs can modulate angiogenesis and increase vessel wall permeability allowing myeloma cells to enter the blood circulation (90, 91). In addition, EVs can protect disseminated cells in the blood circulation from immune attack by providing an immunosuppressive “cover” (92). The presence of EVs also generates a cytokine-enriched niche for myeloma cell survival at extramedullary localizations (40).

The extracellular matrix (ECM) of the BM acts as a scaffold enabling cancer cells to adhere and to interact with its components or with other cells. Treatment with bortezomib and melphalan induces secretion of exosomes containing heparanase that results in degradation of ECM; thus, facilitating myeloma invasion (93). The TME provides a complex network of molecules that can affect the phenotype of PCs. Depending on the stimulus, PCs can acquire a pro-migratory profile that favors cell dissemination or stay in an adherent state allowing engraftment and tumor growth.

## Conclusion

Even though the overall survival and response rate of MM patients have significantly improved due to the introduction of novel therapeutic agents, MM is still an incurable disease. Different studies have shown that the pathophysiology of MM is dynamically supported by strong and dynamic interactions with the surrounding microenvironment. Direct and indirect signaling pathways between malignant plasma cells and the TME can regulate plasma cell adhesion, cellular motility and the generation of new blood vessels. Additional factors such as hypoxia or nutrient-deprivation can further support the invasiveness of single plasma cell clones that finally enter the blood circulation. Cellular and non-cellular contents of the blood can improve the survival of disseminated plasma cells and facilitate the engraftment at distant body sites. Aside, acquisition of secondary mutations selects clones that are no longer dependent on the extrinsic support of the BM. A better understanding of the complex interactions between the TME and MM cells might potentially lead to new therapeutic approaches and result in an improved progression-free survival with reduced risks for disease relapse or extramedullary dissemination. For this reason, the TME is a promising therapeutic target, especially for aggressive, recurrent and/or advanced disease stages. In order to specify the relevance of those therapeutic approaches, future studies must be carried out in a larger and multicenter setting.

## Author Contributions

SF and RR wrote the manuscript. All authors contributed to the article and approved the submitted version.

## Funding

Open access funding provided by University Of Bern.

## Conflict of Interest

The authors declare that the research was conducted in the absence of any commercial or financial relationships that could be construed as a potential conflict of interest.

## Publisher’s Note

All claims expressed in this article are solely those of the authors and do not necessarily represent those of their affiliated organizations, or those of the publisher, the editors and the reviewers. Any product that may be evaluated in this article, or claim that may be made by its manufacturer, is not guaranteed or endorsed by the publisher.
